# Altered adolescents obesity metabolism is associated with hypertension: a UPLC-MS-based untargeted metabolomics study

**DOI:** 10.3389/fendo.2023.1172290

**Published:** 2023-05-09

**Authors:** Zhi-Ping Wu, Wei Wei, Yuan Cheng, Jing-Yi Chen, Yang Liu, Shan Liu, Meng-Die Hu, Heng Zhao, Xiao-Feng Li, Xin Chen

**Affiliations:** ^1^ Department of Epidemiology, School of Public Health, Dalian Medical University, Dalian, China; ^2^ Department of Neurosurgery, Central Hospital of Dalian University of Technology, Dalian, China; ^3^ Institute of Health Science, China Medical University, Shenyang, China

**Keywords:** adolescent obesity, untargeted liquid chromatography-mass spectrometry analysis, hypertension, systolic blood pressure, diastolic blood pressure

## Abstract

**Objective:**

This study aimed to explore the relationship between the plasma metabolites of adolescent obesity and hypertension and whether metabolite alterations had a mediating effort between adolescent obesity and hypertension.

**Methods:**

We applied untargeted ultra-performance liquid chromatography–mass spectrometry (UPLC-MS) to detect the plasma metabolomic profiles of 105 adolescents. All participants were selected randomly based on a previous cross-sectional study. An orthogonal partial least squares- discriminant analysis (OPLS-DA), followed by univariate statistics and enrichment analysis, was used to identify differential metabolites. Using logistic regression for variable selection, an obesity-related metabolite score (OMS, $OMS=\sum_{k=1}^{n}\beta_{n}metabolite{\;}_{n}$ ) was constructed from the metabolites identified, and hypertension risk was estimated.

**Results:**

In our study, based on *P<* 0.05, variable importance in projection (VIP) > 1.0, and impact value > 0.1, we identified a total of 12 differential metabolites. Significantly altered metabolic pathways were the sphingolipid metabolism, purine metabolism, pyrimidine metabolism, phospholipid metabolism, steroid hormone biosynthesis, tryptophan, tyrosine, and phenylalanine biosynthesis. The logistic regression selection resulted in a four-metabolite score (thymidine, sphingomyelin (SM) d40:1, 4-hydroxyestradiol, and L-lysinamide), which was positively associated with hypertension risk (odds ratio: 7.79; 95% confidence interval: 2.13, 28.47; for the quintile 4 compared with quartile 1 of OMS) after multivariable adjustment.

**Conclusions:**

The OMS constructed from four differential metabolites was used to predict the risk of hypertension in adolescents. These findings could provide sensitive biomarkers for the early recognition of hypertension in adolescents with obesity.

## 1 Introduction

Adolescent obesity has reached epidemic proportions worldwide (1, 2). Particularly in low- and middle-income countries, the prevalence of adolescents who are overweight and have obesity is increasing rapidly (3). In China, the number of individuals who are overweight or obese has also increased significantly over the past 30 years. According to a report on nutrition and chronic diseases in Chinese residents in 2015-2019, 11.1% and 7.9% of children and adolescents aged 6-17 years were either overweight or obese, respectively (4). Several studies have demonstrated that obesity in childhood is not only strongly associated with the development of obesity in adulthood but may also increase the incidence of other cardiovascular diseases (5, 6). Baker et al. (7) reported that adolescents with obesity or those that are overweight are at higher risk of developing hypertension compared to normal-weight adolescents. The risk of developing hypertension increases with increasing body mass index (BMI). In addition, hypertension in childhood is a strong predictor of hypertension in adulthood (8). Consequently, the early detection of hypertension in obese children or adolescents and timely preventive measures in high-risk populations can effectively reduce the occurrence of cardiovascular events in adulthood.

Obesity hypertension is generally accepted to be the result of an interaction between genetics and the environment (9). Considering the complexity of hypertension development, an analysis of molecular processes may be “closer” to the disease than genetic markers and more conducive to revealing potential new biomarkers and the pathogenesis of hypertension. Pathophysiological alterations in hypertension are likely to have occurred before measurable elevated blood pressure in the brachial artery of the upper arm (10). When identified, these early physiological or biochemical perturbations will provide a better understanding of hypertensive pathogenesis and offer new opportunities for developing new therapeutic modalities and improving diagnostic methods. In the last decade, metabolomics, which is the qualitative and quantitative assessment of small molecular metabolites (<1500 Da) in bodily fluids, has played an increasingly important role in identifying disease biomarkers (11, 12). The strong association between hypertension and certain dyslipidemia such as cholesterol and triglycerides has been recognized for decades. However, there may also be a link between other metabolic disorders and hypertension, which remains to be discovered. Therefore, newly identified metabolic biomarkers could strengthen the risk prediction models for hypertension.

In our study, we aimed to analyze the differences in metabolic composition associated with obesity and to identify potential metabolic patterns using untargeted ultra-performance liquid chromatography (UPLC)–mass spectrometry (MS) metabolomic techniques based on supervised learning pattern recognition methods. We then explored the relationship between the constructed obesity-related metabolite score and hypertension. We hope to provide scientific evidence for the early identification of susceptibility to adolescent hypertension and for the primary prevention of adult hypertension.

## 2 Materials and methods

### 2.1 Study population

This study was based on a previous cross-sectional study (13), in which random cluster sampling methods were conducted in a randomly selected high school in Shenyang City, Liaoning Province, China. The cross-sectional study included three components: an administered questionnaire, health examination, and blood sample collection.

In this study, we randomly selected 105 adolescents aged 15 to 17 years (53 boys and 52 girls) from a previous cross-sectional study, in which the adolescents were included with complete anthropometric and biochemical information. The exclusion criteria were any other chronic disease, autoimmune diseases, or taking any medication. Normal-weight (BMI<85^th^ percentile for age and sex), overweight (BMI ≥85^th^ percentile but<95^th^ percentile for age and sex), and obese (BMI ≥95^th^ percentile for age and sex) groups were defined from Chinese population data (14). The study was approved by the ethics committee of Dalian Medical University. All participants and their parents gave informed consent.

### 2.2 Questionnaire administered and health examination

Standardized training was administered to the interviewers before the survey began. Only trained and qualified interviewers conducted the survey, using a face-to-face method at the school. Structured questionnaires were used to collect data on demographic information (age, sex, ethnicity, and the number of household sizes), emotional components (self-evaluation, family evaluation, friends evaluation, and self-control ability), and life sections (sleep status, living conditions, and physical activity). The completed surveys were reviewed by two independent quality control interviewers.

Weight, height, and blood pressure were performed by trained professionals following standard-based protocols (referenced to GB/T26343). According to the Chinese Reference on Blood Pressure in Children and Adolescents, hypertension was defined as systolic/diastolic blood pressure (SBP/DBP) ≥95^th^ percentile for age, sex, and height (15).

### 2.3 Blood sample collection and preparation

Fasting blood samples were drawn from all the adolescents after a 12-hour fast. The samples were added to an ethylenediaminetetraacetic acid (EDTA) anticoagulation tube, mixed upside down 8–10 times, and centrifuged at 2,500 rpm for 10 minutes at room temperature for plasma separation. The extracted plasma sample (40 μL) was transferred to a 2 mL Eppendorf (EP) tube. Ice-cold methanol was added (160 μL) to remove protein. The centrifuge tube was vortexed at maximum speed for 10 s and centrifuged at 13,500 rpm for 15 min. Supernatants (80 μL) were transferred to liquid chromatography (LC) vials and stored at -80°C until the ultra-performance liquid chromatography (UPLC)–mass spectrometry (MS) analysis. In addition, triglycerides (TG), cholesterol (TC), high-density lipoprotein cholesterol (HDLC), and low-density lipoprotein cholesterol (LDLC) were measured by the Shengjing Hospital of China Medical University using a Hitachi 7600 clinical analyzer (Hitachi, Tokyo, Japan).

### 2.4 Untargeted ultra-performance liquid chromatography–mass spectrometry metabolomics analysis

**Figure 1:**
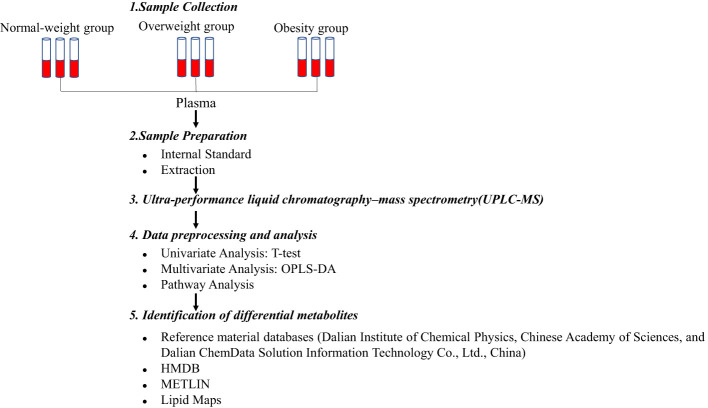
Study procedure for the untargeted metabolomics analysis.

Metabolomics analysis was conducted using standard operating procedures for untargeted metabolomics analysis (**Figure 1**). A UPLC system (Thermo Fisher Scientific, Waltham, MA, USA) was equipped with an ACQUITY BEH C8 column (1.7μm, 2.1×100nm, Waters Corp, Milford, USA). The binary gradient elution system consisted of (A) water and (B) 95% acetonitrile, which both contained 0.1% formic acid. Separation was performed by using the following: 0 min, 5% B; 1 min, 5% B; 24 min, 98% B; 27.5 min, 98% B; 27.6 min, 5% B; 31 min, 5% B. The flow rate was 0.35 mL/min. The injection volume was 10μL. All analyses were performed at 4°C.

MS analysis was conducted on a Q-Exactive mass spectrometer outfitted with a heated electrospray ionization source (Thermo Fisher Scientific, Waltham, MA, USA) with positive and negative ion modes. Data acquisition was performed in full scan mode (m/z ranges from 75 to 1,000) combined with data-dependent acquisition (DDA) mode (full MS/dd-MS2 scans). The parameters for full scan mode and DDA mode were as follows: resolution, 70,000/17,500; automatic gain control (AGC) target, 3e6/1e5; normalized collision energy (NCE): 20/40ev. The following were the parameters of the MS: spray voltage, 3.0kV (positive), 3.0kV (negative); evaporation temperature, 320°C; sheath gas flow rate, 35 arbitrary units; auxiliary gas flow rate, 8 arbitrary units; capillary temperature, 350 °C; S-lens radio frequency (RF): 50.6.

The quality control samples were performed by mixing an equivalent portion of supernatant (10 μL). They were injected periodically (every 12 samples) throughout the analysis run to provide a set of data that can be used for analytical repeatability.

### 2.5 Metabolomics data pre-processing and analysis

Metabolomics data were acquired using Xcalibur 2.1 software (Thermo Fisher Scientific), which generated a data matrix consisting of retention time (RT), mass-to-charge ratio (m/z) values, and peak intensity. OSI-SMMS (One-step Solution for Identification of Small Molecules in Metabolomics Studies) was used for peak annotation. Metabolites were identified by searching major metabolite databases, including reference material databases (Dalian Institute of Chemical Physics, Chinese Academy of Sciences, and Dalian ChemData Solution Information Technology Co., Ltd., China), HMDB (http://www.hmdb.ca/), METLIN (http://metlin.scripps.edu/), and Lipid Maps (https://lipidmaps.org/).

For the univariate statistical analyses, we used volcano plots to visualize metabolite differences between groups. A fold change of 1.5 was used as the threshold for up-regulated and down-regulated metabolites. Multivariate analysis was performed using orthogonal partial least-squares-discriminant analysis (OPLS-DA) to differentiate the obesity and normal-weight groups. In this study, we were more concerned with exploring the metabolite alterations between the normal-weight and obese groups, because obesity is a serious metabolic disease whose pathophysiological mechanisms are very different from those of normal-weight metabolism; we believe that the comparison between the normal-weight group and obese group can better reveal the characteristics and mechanisms of metabolic abnormalities in obesity. We evaluated the model performance using model parameters R2Y (interpretability of the model) and Q2 (predictability of the model), obtained from 7-fold cross-validation. In addition, the permutation test was applied to evaluate the model using the regression line, and the Y-axis intercept values (R2 and Q2) were used to measure whether the model was overfitted. If the intercept of Q2 was less than zero, then the model was valid (16). The preliminary criteria for identifying differential metabolites were *P*< 0.05 from the Student’s *t*-test and variable importance in projection (VIP) >1.0 in the OPLS-DA model. In addition, metabolic pathway analysis was performed using the Kyoto Encyclopedia of Genes and Genomes (KEGG), which identified metabolic pathways with a significant important value on metabolites (impact value > 0.1). We further used restricted cubic spline curves with three knots to visualize the dose–response relationship between the BMI and metabolites and box plots to visualize the metabolite values with statistically significant differences between the groups.

### 2.6 Statistical analysis

We calculated the mean (standard deviation) of the continuous variables and the number (percentage) of the categorical variables. One-way ANOVA and the chi-square test were used to compare the continuous and categorical variables, and Bonferroni correction was used for multiple group comparisons. Natural log transformation was performed on all the selected differential metabolite values, which were then converted to the standard normal distribution. We evaluated the relationships between the selected differential metabolites and biochemical indicators with the selected differential metabolites using the Spearman partial correlation coefficient. We used logistic regression to construct a parsimonious model that effectively represented the metabolic elements associated with adolescent obesity. These were used to create the obesity-related metabolite score (OMS), as follows:


$$OMS=\beta_{1}metabolite_{1}+\beta_{2}metabolite_{2}+\beta_{3}metabolite_{3}+\ldots +\beta_{n}metabolite_{n}$$

where $\beta_{n}$ was the corresponding regression coefficient for metabolite (n), $metabolite_{n}$ was the Z-score for metabolite (n), and n was the amount of selected differential metabolites. Next, we evaluated the association between the OMS and hypertension risk of the adolescents. The scores were assessed in quartiles (based on controls) using unconditional logistic regression after multivariable adjustment. We also used stepwise multiple linear regression to evaluate the relationship between the OMS and SBP/DBP. Finally, we used a mediation effect, which divided the total effect into the direct effect (i.e., independent OMS) and indirect effect (i.e., mediated OMS), to estimate the degree to which the selected differential metabolites (continuous OMS) could account for the relationship between obesity and the risk of developing hypertension in adolescents. To further explore potential confounders, we used sensitivity analyses to estimate the mediation effect of the OMS by adjusting for sex, age, ethnicity, and lipid indicators.

## 3 Results

### 3.1 Baseline information of the study population

In total, 105 adolescents who fulfilled the inclusion and exclusion criteria were included in this study and were assigned to normal-weight (n = 53), overweight (n = 34), and obesity (n = 18) groups, as presented in **Table 1**. Briefly, adolescents in the obesity group had significantly higher weight, BMI, SBP, DBP, and lipids (LDLC, CHOL, and TG) after Bonferroni correction (*P*<0.05/3). We also found that DBP in the overweight group was the lowest compared to the normal-weight and obese groups (*P<*0.05/3). In addition, there was no significant statistical difference between the groups in terms of age, sex, and height (*P*>0.05).

**Table 1:** Baseline characteristics of the studied population.

Parameter	NW Group (n=53)	OW Group (n=34)	OB Group (n=18)	χ^2^/F	*^a^p*-Value
Age(years)	16.06(0.31)	16.06(0.24)	16.26(0.54)	0.001	0.999
Male(%)	25(0.48)	17(0.50)	11(0.62)	1.049	0.592
Weight, kg	57.29(6.15)	70.31(7.43)	87.22(11.99)	104.055	<0.001
Height, m	1.68(0.09)	1.67(0.08)	1.67(0.09)	0.492	0.613
BMI, kg/m^2^	20.16(0.88)	25.24(1.06)	31.19(2.88)	410.184	<0.001
SBP(mmHg)	121.42(12.27)	126.32(12.26)	138.14(15.07)	11.541	<0.001
DBP(mmHg)	71.87(8.59)	69.22(7.64)	76.22(10.20)	3.917	0.023
LDLC(mmol/L)	2.17(0.84)	2.20(0.50)	2.66(0.80)	3.109	0.049
HDLC(mmol/L)	1.46(0.33)	1.24(0.28)	1.24(0.28)	6.590	0.002
CHOL(mmol/L)	3.90(0.65)	3.83(0.58)	4.43(0.98)	4.876	0.010
TG(mmol/L)	0.82(0.34)	0.87(0.37)	1.43(0.91)	10.933	<0.001

NW, normal weight; OW, overweight; OB, obesity; BMI, body mass index; SBP, systolic blood pressure; DBP, diastolic blood pressure; LDLC, low-density lipoprotein cholesterol; HDLC, high-density lipoprotein cholesterol; CHOL, cholesterol; TG, triglycerides.

Continuous variables are presented as mean (standard deviation) and compared using one-way ANOVA, whereas categorical variables are presented as n(%) and compared by chi-square test. Bonferroni correction was used for multiple group comparisons.

^a^p-Value from one-way ANOVA (chi-square test).

### 3.2 Untargeted metabolomics analysis

**Figure 2:**
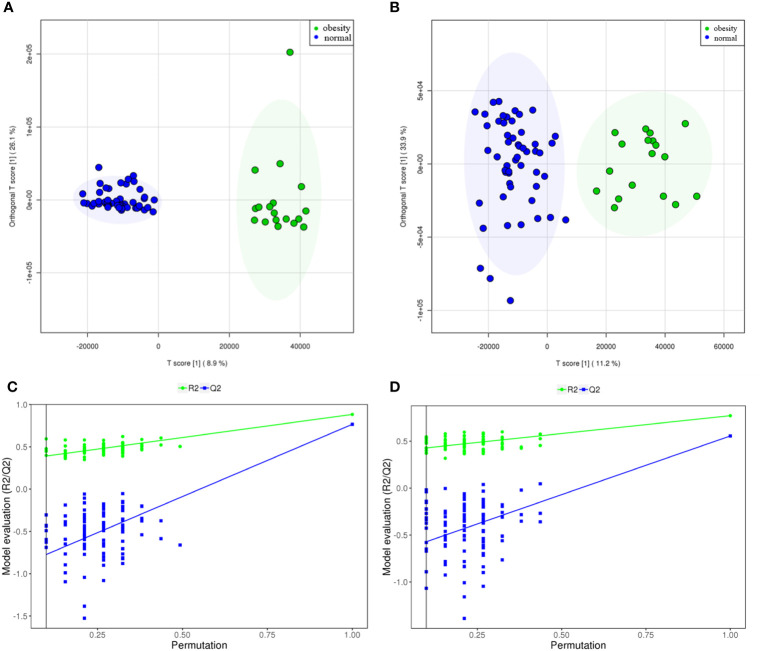
The metabolites from adolescents with obesity were compared to those from adolescents with normal weight. The scoring plot of the OPLS-DA model and the permutation test plot [positive ion mode **(A, C)**, and negative ion mode **(B, D)**. The permutation test plot can be explained as follows: the vertical axis represents the R2/Q2 value for the original model (far to the right) and of the cluster of Y-permuted models further to the left, which means that the positive ion mode was R2Y(cum)= 0.93, Q2Y(cum)= 0.57, while the negative ion mode was R2Y(cum)= 0.89, Q2Y(cum) =0.58.

**Figure 3:**
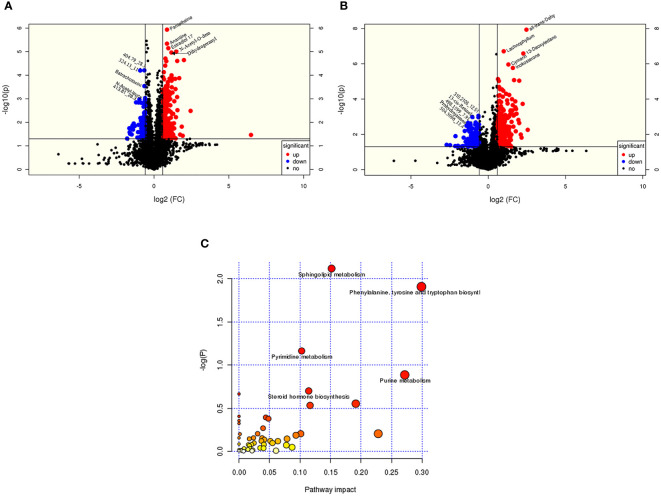
The metabolites from adolescents with obesity were compared to those from adolescents with normal weight. Positive ion **(A)** and negative **(B)** ion mode differential metabolite volcano map. The abscissa represented the fold change of the group compared to each substance (base 2 logarithm). The ordinate represented the P-value (base 10 logarithm), and pathway analysis **(C)** showed that differential metabolites are concentrated in sphingolipids, purines, and pyrimidine, and the biosynthesis of amphetamine acid, tyrosine, tryptophan, and steroid hormones.

According to the OSI/SMMS software, 705 differential metabolites were identified. In the ESI-positive ion modes, 477 differential metabolites were identified in adolescents of normal weight and those with obesity. In the ESI-negative ion modes, 415 differential metabolites were identified (**Supplementary Table 1**). The OPLS-DA score plot obtained from the multivariate statistical analysis (**Figures 2A, B**) demonstrated that the normal-weight group and obesity group could be clearly distinguished. Subsequently, the results of the permutation test of the OPLS-DA model (**Figures 2C, D**) revealed that the positive ion mode was R2Y(cum) = 0.93, Q2Y(cum) = 0.57, while the negative ion mode was R2Y(cum) = 0.89, Q2Y(cum) = 0.58. These parameters demonstrated that the model was robust in terms of predictive performance without overfitting. In addition, we used the volcano plot to visualize the distribution of the differential metabolites between the groups (**Figures 3A, B**). Finally, the metabolic pathway analysis indicated that 705 metabolites were involved in 50 pathways. Through an impact-value screen (impact value > 0.1), nine pathways were selected for further analysis. The bubble plot (**Figure 3C**) also revealed that differential metabolites were predominantly enriched in the pathways of sphingolipid metabolism, purine metabolism, pyrimidine metabolism, steroid hormone biosynthesis, tryptophan biosynthesis, tyrosine biosynthesis, phenylalanine biosynthesis, inositol phosphate metabolism, and glycerophospholipid metabolism.

### 3.3 Identification of differential metabolites

Based on *P<*0.05, VIP > 1.0, and impact value >0.1, we identified a total of 12 differential metabolites (including seven down-regulated and five up-regulated differential metabolites, **Table 2**). These selected differential metabolites presented a dose–response relationship with BMI by restricted cubic spline curves with three knots, and they were statistically significant differences in at least one group in a two-by-two comparison by boxplots (*P*<0.05/3) (**Supplementary Figures 1**, **2**).

**Table 2:** Important plasma differential metabolites between obesity and normal-weight group.

Metabolites	Ion Mode	Pathways	Changing Trend	Retention Time	Mass-to-charge ratio
L-Tryptophan	POS	Phenylalanine, tyrosine, and tryptophan biosynthesis	Down	11.30	246.12
PC (15:0/16:0)	NEG	Phospholipid metabolism	Down	25.38	778.56
PC 38:6	NEG	Phospholipid metabolism	Up	27.56	850.56
Carbamoyl phosphate	NEG	Purine metabolism	Down	6.28	421.93
Thymidine	POS	Pyrimidine metabolism	Up	9.65	523.14
13-cis-Retinol	NEG	Related to lipid metabolism	Down	28.22	857.67
Shikimate	NEG	Phenylalanine, tyrosine, and tryptophan biosynthesis	Down	7.19	233.07
SM d40:1	POS	Sphingolipid metabolism	Down	26.11	787.67
5’-Phosphoribosylglycinamide	NEG	Steroid hormone biosynthesis	Up	6.99	631.12
4-Hydroxyestradiol	NEG	Steroid hormone biosynthesis	Up	9.83	621.35
L-Lysinamide	NEG	Purine metabolism	Down	7.88	166.09
DHEA Sulfate	NEG	Steroid hormone biosynthesis	Up	9.81	367.16

POS, positive; NEG, negative; Up, up-regulated; Down, down-regulated.

**Figure 4:**
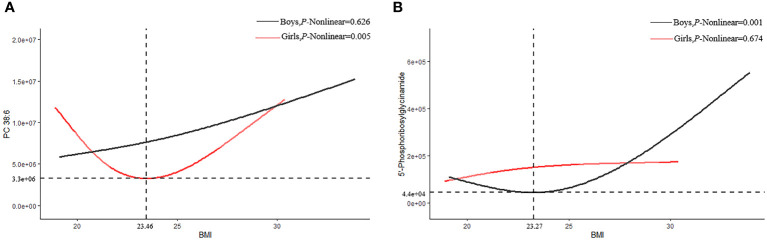
Restricted cubic splines of PC38:6 and 5'-Phosphoribosylglycinamid. **(A)** PC 38:6 **(B)** 5-phosphoribosylglycinamide.

Interestingly, we also found that PC 38:6 and 5´-phosphoribosylglycinamide had U-shaped associations with BMI, in which the inflection points of BMI were 23.07 kg/m^2^ and 21.97 kg/m^2^, respectively. Before the inflection point, PC 38:6 and 5´-phosphoglycinamide were negatively correlated with BMI; in contrast, they were positively correlated with BMI after the inflection point. P38:6 decreased before BMI reached 23.46 kg/m^2^ and then increased in the female participants (*P* for non-linear=0.005). However, this trend was not found among the male participants (*P* for non-linear=0.626). In the male participants, 5´-phosphoribosylglycinamide decreased before BMI reached 23.27 kg/m^2^ and then increased (*P* for non-linear=0.001). However, this trend was not found among the female participants (*P* for non-linear=0.674, **Figure 4**).

### 3.4 Association between metabolite scores and hypertension in adolescent obesity

**Figure 5:**
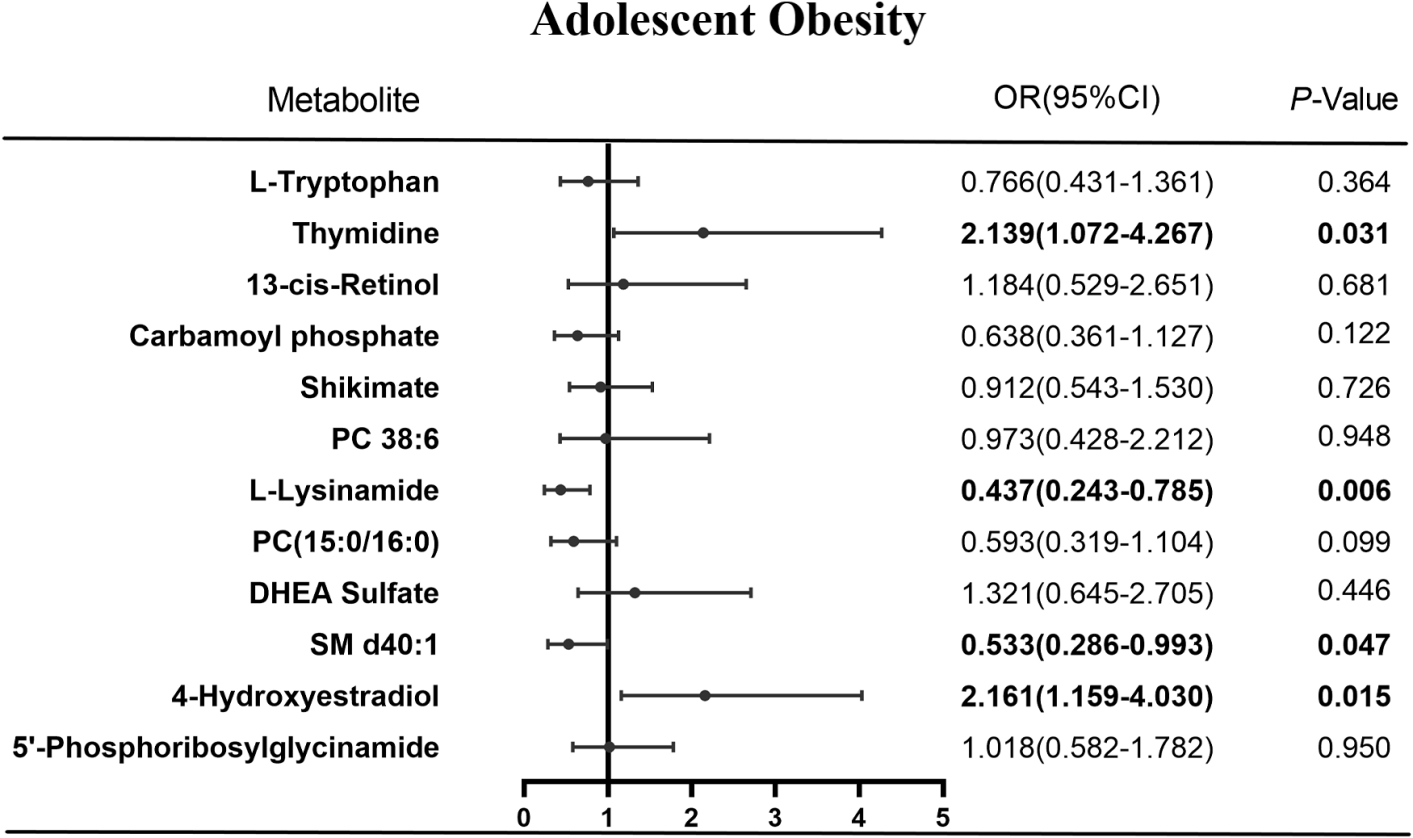
Results of logistic regression of metabolites and obesity.

From the 12 differential metabolites, the logistic regression algorithm selected a parsimonious model of four metabolites, namely thymidine (OR=2.14, 95% CI=1.07-4.27, *P*=0.031), SM d40:1 (OR=0.53, 95% CI=0.29-0.99, *P*=0.047), 4-hydroxyestradiol (OR=2.16, 95% CI=1.16-4.03, *P*=0.015), and L-lysinamide (OR=0.44, 95% CI=0.24-0.79, *P*=0.006) (**Figure 5**). The OMS constructed from these four metabolites was positively associated with hypertension risk. In developing hypertension risk models adjusted for age, sex, and ethnicity, the odds ratio (95% CI) was 7.79 (2.13-28.47) for quintile 4 compared with quartile 1 of OMS. The linear trend across the quartiles was significant (*P*-trend=0.017). There was a 48% higher odd ratio of developing hypertension after multivariate adjustment associated with each SD increase in OMS when the scores were assessed continuously. Further adjustment of the lipid markers diminished this correlation, and the correlation vanished in quartile 3 compared with quartile 1 of the OMS (OR=2.83, 95% CI=0.68-11.84, **Table 3**).

**Table 3:** Associations of the obesity-related metabolite score with hypertension risk.

	Obesity-related metabolite score (OMS)^a^ -Odds ratio (95% CI)					Per SD increase in OMS	*P-*trend
	Q1	Q2		Q3	Q4		
Model 1^b^	1.00(ref)	4.07(1.21,13.69)		3.86(1.14,13.14)	7.21(2.02,25.82)	1.47(1.11,1.94)	0.020
Model 2^c^	1.00(ref)	4.72(1.32,16.93)		4.01(1.16,13.82)	7.79(2.13,28.47)	1.48(1.12,1.96)	0.017
Model 3^d^	1.00(ref)	5.18(1.20,22.38)		2.83(0.68,11.84)	5.52(1.25,24.47)	1.34(0.97,1.84)	0.095

^a^The metabolite score included four obesity-related metabolites (thymidine, SM d40:1, 4-hydroxyestradiol, L-lysinamide).

Model 1^b^ adjusted for sex.

Model 2^c^ Model 1+ adjusted for age and ethnicity.

Model 3^d^ Model 2+adjusted for triglyceride, cholesterol, HDLC, LDLC.

Next, the stepwise multiple linear regression showed that after adjusting for age, sex, ethnicity, triglyceride, cholesterol, HDLC, LDLC, and OMS, the factors that independently influenced SBP were sex (β=-12.74,95% CI:-17.29,-8.20, *P*=0.001), triglyceride (β=7.39, 95% CI: 2.09-12.68, *P*=0.007), and OMS (β=1.88, 95% CI: 0.38-3.38, *P*=0.015, **Supplementary Table 2**), and the factor that independently influenced DBP was triglyceride (β=4.30, 95% CI:0.60-8.00, *P*=0.023, **Supplementary Table 3**); however, a similar association was not observed between OMS and DBP (*P*>0.05).

### 3.5 Mediation effect of obesity and hypertension risk

Compared with the normal-weight adolescents, the adolescents that were obese/overweight had a 23% higher hypertension risk (95% CI:1.09,1.39, **Table 4**). Approximately 47.5% of the association was mediated with OMS (OR: 1.18, 95%CI: 0.84,1.64). After adjusting for indicators of lipids, the relationship between obesity and the risk of developing hypertension was diminished, where the total effect of obesity and risk of developing hypertension was 1.19 (95%CI:1.02,1.37), and the indirect effect was 1.15 (95%CI: 0.80, 1.65). The OMS accounted for a comparable extent (48.6%) of the relationship between obesity and the risk of developing hypertension in the adjusted model.

**Table 4:** Association of obesity with hypertension risk overall (total), mediated by OMS^a^ (indirect) and independent of OMS (direct).

	Total effect	Direct effect	Indirect effect	% Mediated
	OR (95% CI)	OR (95% CI)	OR (95% CI)	
Model 1^b^	1.23(1.09,1.39)	1.19(1.04,1.36)	1.17(0.84,1.62)	45.9%
Model 2^c^	1.23(1.09,1.39)	1.19(1.04,1.36)	1.18(0.84,1.64)	47.5%
Model 3^d^	1.19(1.02,1.37)	1.15(0.98,1.36)	1.15(0.80,1.65)	48.6%

OMS, Obesity-related metabolite score; ^a^the metabolite score included four obesity-related metabolites (thymidine, SM d40:1, 4-hydroxyestradiol, L-lysinamide).

Model 1^b^ adjusted for sex.

Model 2^c^ Model 1+ adjusted for age and ethnicity.

Model 3^d^ Model 2+adjusted for triglyceride, cholesterol, HDLC, LDLC.

## 4 Discussion

The present study proposed that the OMS based on the four representative metabolites (thymidine, SM d40:1, 4-hydroxyestradiol, L-lysinamide) was associated with hypertension risk, and it was evaluated to potentially mediate the association between obesity and the risk of developing hypertension. These observational data demonstrated momentous mechanistic insights into how obesity might contribute to the risk of developing hypertension in adolescents through dysregulation of the lipid metabolism.

Previous studies have estimated the effects of obesity on plasma metabolites. An untargeted metabolomic study of 27 obese Caucasian adolescents and 15 sex- and age-matched normal-weight adolescents concluded that lipids and amino acids were the most important chemical classes among the different metabolites (17). Habibi et al. (18) reported that abnormalities of the sphingolipid and phospholipid metabolism were strongly associated with the lipid metabolism. Our findings showed that the sphingolipid metabolism was significantly abnormal in the obese adolescents, with their sphingolipid levels significantly lower than in the normal-weight/overweight groups, which is consistent with the results of a study in Chinese adolescents (19) but inconsistent with the study of Qatari adults (20). Purine metabolism and pyrimidine metabolism, which are highly related to the lipid metabolism, were also important metabolomic alterations in obese adolescents. This observation was consistent with the limited data available for children and adolescents (17, 21). The abnormalities of the aromatic amino acid metabolism resulted in lower levels of tyrosine, phenylalanine, and tryptophan in the obese adolescents compared to the normal-weight adolescents in this study, with significant trends in metabolites associated with the shikimate pathway in particular. However, several previous studies (22–24) have reported that elevated circulating Tyr and Phe levels have been frequently found in obesity, insulin resistance, or diabetes in humans. This may be explained by abnormalities in gut function, microbial populations, and diet structure in obese patients, resulting in metabolite alterations. In addition, we also found elevated levels of steroid hormones in the obese adolescents, which was consistent with several previous studies conducted on children and adolescents (17, 25, 26). Thus, in this study, the lipid metabolism and amino acid metabolism were proposed to be the most important metabolic pathways in obese adolescents.

Thymidine was found to be enriched in the pyrimidine metabolic pathway in this study. Gelpi et al. (27) demonstrated that prior exposure to thymidine analogs increased the risk of hypertension. Not only may thymidine analogs be irreversibly and deleteriously associated with visceral adipose tissue (VAT) accumulation, but the induced oxidative stress may lead to mild mitochondrial dysfunction in adipocytes (28). VAT was an independent risk factor for cardiovascular and metabolic diseases (29, 30). Our study showed that thymidine was up-regulated in obese adolescents and was associated with hypertension. The accumulation of VAT in obese adolescents stimulated the elevated thymidine level. Accumulation of VAT has been frequently found in the renin–angiotensin–aldosterone system (RAAS), free fatty acids, and insulin resistance (31), which were known risk factors for hypertension and abnormalities in the lipid metabolism.

SMs are components of lipid rafts, ceramide precursors, and other sphingolipid metabolites involved in the signaling pathway (32). Ceramide, which is produced in the endoplasmic reticulum, serves a central role in the sphingolipid metabolism (33). Ceramide synthases transfer a fatty acid from acyl-CoA to the SM scaffold to form dihydroceramide. Then, dihydroceramide desaturases (DES1 and DES2) are required to produce ceramide (34). In addition to its structural role in the cell membrane, ceramide elicits an initial cellular response to lipid burden during physiological or nutritional stress. In the long term, ceramide contributes to an understanding of the pathophysiology of type 2 diabetes, hypertension, and atherosclerosis. Notably, circulating ceramide levels have become a predictive biomarker for cardiometabolic complications (35–37). Our study showed that SM d40:1 was associated with hypertension in adolescents. This may be a result of elevated circulating lipid levels in adolescents with obesity leading to the accumulation of fat deposits in the vascular lumen and ectopic formation of ceramides in the vascular endothelial cells. Ceramide disrupts the interaction between protein phosphatase 2A (PP2A) and 2 of PP2A (I2PP2A), which results in an increased association between PP2A and eNOS (38). This impairs endothelium-dependent vasorelaxation and exacerbates vasoconstriction.

L-lysinamide, one of the products of L-lysine metabolism, was enriched in the purine metabolic pathway. Alterations in the lysine metabolism were strongly associated with hypertensive patients on low- or high-sodium diets (39–41). A urine metabolomics study in the Framingham cohort showed that high levels of lysine were responsible for the protective effect of hypertension (42). Lewis et al. (43) concluded that purines and purine products were closely associated with right ventricular–pulmonary vascular dysfunction. Several previous studies also demonstrated that the correlation between the final product of the purine metabolism (i.e., uric acid) and hypertension had been sufficiently described in children and adolescents, with mechanisms that primarily implicated oxidative stress and intracellular urate activity (44–46). Our study showed that L-lysinamide was associated with hypertension in adolescents. On the one hand, due to the high intake of purine food in adolescents with obesity, the purine metabolism was dysfunctional and induced oxidative stress-promoted endothelial dysfunction, vascular remodeling, and inflammation, resulting in vascular injury. On the other hand, due to abnormal sphingolipid metabolism, there was obvious insulin resistance in these obese adolescents, leading to abnormal purine metabolism and high uric acid (47, 48).

4-hydroxyestradiol is the most active of the endogenous estrogen metabolites. Adler et al. (49) reported that estrogen exposure increased endothelial-dependent vasodilation in lean healthy women. Activation of estrogen receptors α and β in the endothelial cells causes the production of endothelial nitric oxide synthase (eNOS) and the expression of genes encoding eNOS (50). Khan et al. (51) reported that abnormal estradiol concentrations may alter the normal function of insulin, which could lead to insulin resistance in patients. Estradiol deficiency and RAAS imbalance increased the incidence of hypertension, arrhythmia, and heart failure (52). Our study showed that 4-hydroxyestradiol was up-regulated in adolescents with obesity and was associated with hypertension. An increase in 4-hydroxyestradiol may prevent the effective stimulation of endothelial cells, resulting in a loss of intravascular homeostasis and vasoconstriction.

The lipid profile of obesity identified in this study has previously been found to predict the development of hypertension risk, diabetes, and insulin resistance (53–55). Consistent with prior studies, our findings demonstrate that the OMS constructed from obesity-based differential metabolites, three of which were well-distributed in the lipid metabolism (thymidine, SM d40:1, L-lysinamide), was strongly correlated with developing hypertension risk and positively correlated with systolic blood pressure, but it did not demonstrate a similar relationship with diastolic blood pressure, which was also an interesting finding. The mediated analysis further illustrated the effect of metabolomic alterations related to obesity in the development of hypertension, with the OMS accounting for approximately 48.6% of the correlation between obesity and the risk of developing hypertension. Dysregulation of the lipid metabolism may be an important mechanism by which obesity increases the risk of developing hypertension. Thus, it is important to find early sensitive biomarkers in obese adolescents that can identify hypertension. In the future, it would be interesting to explore and validate whether early intervention in adolescents with obesity may be a useful way to reduce the occurrence of hypertension.

The present study also had several limitations. First, the main limitation of the present study was the cross-sectional study, which could not derive a clear causal relationship between obesity-related metabolite scores and the risk of developing hypertension in adolescents. Therefore, the present study needs to be validated in other independent cohort studies. Second, alterations in some metabolites (e.g., lipids, amino acids, and steroid hormones) were susceptible to age-related effects. This can result in the deviation of the early identification of metabolic markers of hypertension. Third, dietary structure alterations also influence the expression of metabolites, especially high-fat diets, but the relevant information was inadequate. Therefore, our findings require targeted metabolomics to validate the effect of the gut microbiota on the metabolites. Fourth, all the adolescents in this study were from Shenyang, China, with a limited sample size. Therefore, our findings may not be applicable to the whole Chinese population. Finally, a population-based independent prospective cohort may be needed to verify the results of our predictive model.

## 5 Conclusions

In conclusion, it was identified that thymidine, SM d40:1, 4-hydroxyestradiol, and L-lysinamide in adolescents with obesity were associated with hypertension. These four differential metabolites combined might be also used to predict the risk of developing hypertension. Obesity may increase the hypertension risk through the dysregulation of the lipid metabolism. These findings can provide sensitive biomarkers for the early recognition of hypertension in obese adolescents and scientific evidence for the primary prevention of hypertension in adults.

## Data availability statement

All datasets were included in the article/**Supplementary Material** in this study. Further used and analyzed can be available from the corresponding author upon reasonable request.

## Ethics statement

The studies involving human participants were reviewed and approved by the ethics committee of Dalian Medical University. Written informed consent to participate in this study was provided by the participants’ legal guardian/next of kin.

## Author contributions

Z-PW, WW, X-FL, and XC conceived the study. ZW, WW, YC, J-YC, SL, M-DH, and HZ collected data for all adolescent clinical information. Z-PW, XC, WW, and X-FL performed the statistical analyses. XC, WW, and X-FL supervised the study. Z-PW completed the first draft of the manuscript. All authors contributed to the article and read and approved the submitted final manuscript.
