# Supplementary material for: Altered adolescents obesity metabolism is associated with hypertension: a UPLC-MS-based untargeted metabolomics study
Source: Front Endocrinol (Lausanne). 2023 May 9;14:1172290. doi: 10.3389/fendo.2023.1172290 (PMC10203610; doi:10.3389/fendo.2023.1172290)
Supplement: Supplementary file 1 [file DataSheet_1.docx]

Supplementary Material

**Supplementary Table 1** Summarize of metabolic differences in adolescents with different constitution states.

| **Ion mode** | **Metabolites** | **OB vs NW** | **OW vs NW** | **OB\|OW vs NW** |
| --- | --- | --- | --- | --- |
| POS | Differential metabolites (Up/ Down) | 477(324/153) | 330(239/91) | 398(190/208) |
|  | Known metabolites (Up/ Down) | 347(246/101) | 246(183/63) | 279(153/126) |
| NEG | Differential metabolites (Up/ Down) | 415(239/176) | 183(115/68) | 271(97/174) |
|  | Known metabolites (Up/ Down) | 123(60/63) | 27(13/14) | 68(15/53) |

POS, positive; NEG, negative; Up, upregulated; Down, downregulated; OB, obesity; OW, overweight； NW, normal-weight.

**Supplementary Table 2** Multiple linear regression analysis of SBP as dependent variable.

| **Parameter** | **β** | **SE** | **β(95%CI)** | **t** | ***P*** | ***F*** |
| --- | --- | --- | --- | --- | --- | --- |
| Constants | 146.20 | 3.62 | **-** | 40.40 | 0.001 | F(3,101)=16.428,  *P*<0.001 |
| Sex | -12.74 | 2.29 | (-17.29,-8.20) | -5.56 | 0.001 |  |
| Triglyceride | 7.39 | 2.67 | ( 2.09,12.68 ) | 2.77 | 0.007 |  |
| OMS | 1.88 | 0.76 | ( 0.38,3.38 ) | 2.48 | 0.015 |  |

SBP, systolic blood pressure; OMS, Obesity-related metabolite score. Take SBP as the dependent variable, the stepwise multiple linear regression showed that after adjusting for age, sex, ethnicity, triglyceride, cholesterol, HDLC, LDLC, and OMS, the factors that independently influenced SBP were sex(*β* =-12.74, 95% CI:-17.29,-8.20, *P*=0.001), triglyceride (*β* = 7.39, 95% CI: 2.09,12.68, *P*=0.007), and OMS (*β* = 1.88, 95% CI: 0.38,3.38, *P*=0.015).

**Supplementary Table 3** Multiple linear regression analysis of DBP as dependent variable.

| **Parameter** | **β** | **SE** | **β(95%CI)** | **t** | ***P*** | ***F*** |
| --- | --- | --- | --- | --- | --- | --- |
| Constants | 72.50 | 0.90 | **-** | 80.22 | 0.001 | F(1,103)=5.315,  *P*=0.023 |
| Triglyceride | 4.30 | 1.87 | (0.60,8.00) | 2.31 | 0.023 |  |

DBP, diastolic blood pressure; Take DBP as the dependent variable, the stepwise multiple linear regression showed that after adjusting for age, sex, ethnicity, triglyceride, cholesterol, HDLC, LDLC, and OMS, the factors that independently influenced DBP were triglyceride (*β* = 4.30, 95% CI: 0.60, 8.00, *P*=0.023).


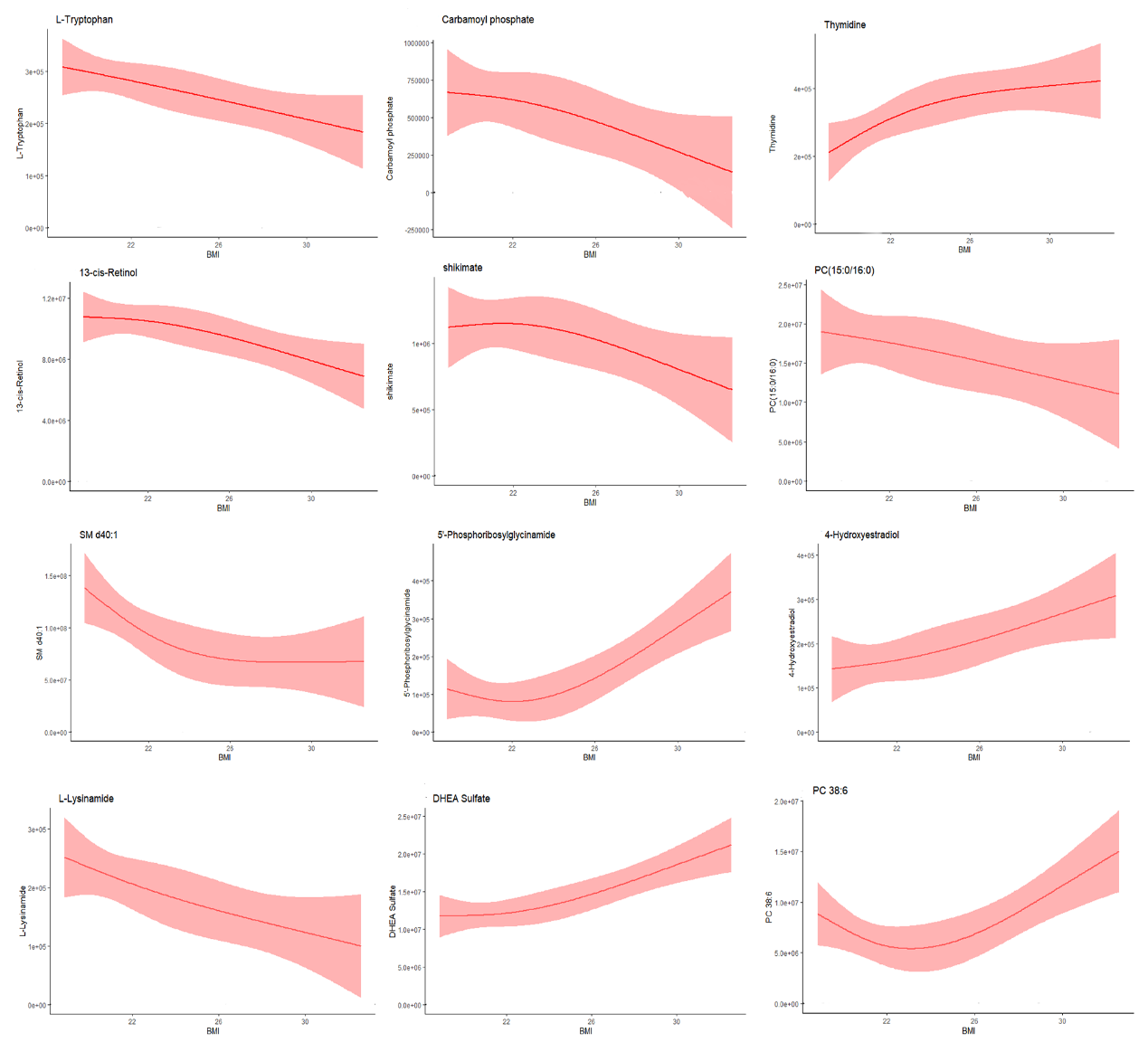
**Supplementary Figure 1** Restrictive cubic spline plot of 12 metabolites and BMI


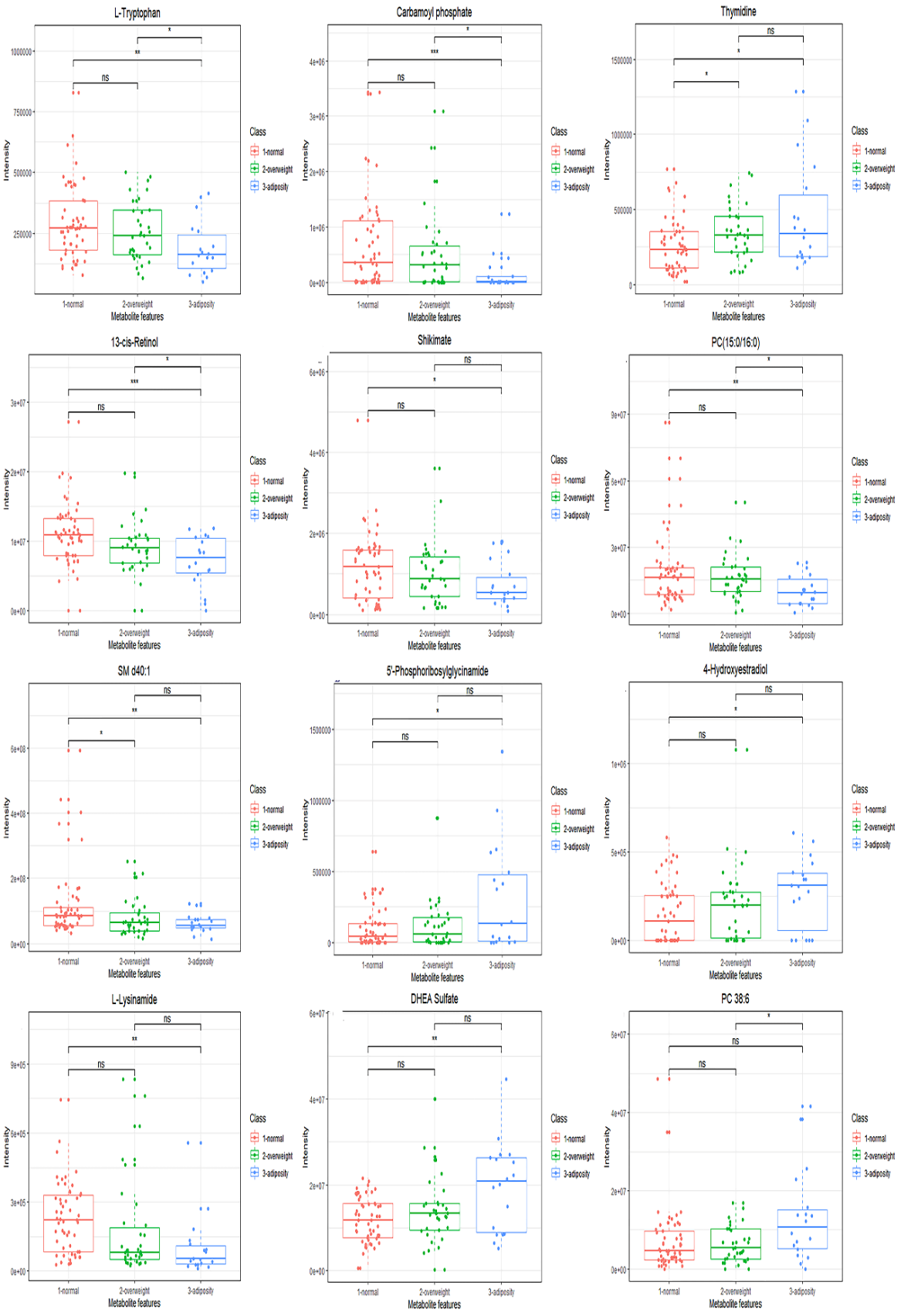


**Supplementary Figure 2** Boxplots of selected metabolites with significantly different concentrations between NW (red), OW (green), and AD (blue). ns: p > 0.05, *: p <= 0.05, **: p <= 0.01, ***: p <= 0.001.
